# Traceable Quantum Steganography Scheme Based on Pixel Value Differencing

**DOI:** 10.1038/s41598-019-51598-8

**Published:** 2019-10-22

**Authors:** Jia Luo, Ri-Gui Zhou, GaoFeng Luo, YaoChong Li, GuangZhong Liu

**Affiliations:** 1 College of Information Engineering, Shanghai Maritime University, Shanghai, 201306 China; 2 Research Center of Intelligent Information Processing and Quantum Intelligent Computing, Shanghai, 201306 China; 3 College of Information Engineering, Shaoyang University, Shaoyang, 422000 China

**Keywords:** Quantum information, Theoretical physics

## Abstract

A novel and traceable quantum steganography scheme based on pixel value differencing (PVD) is proposed. In the proposed scheme, a quantum cover image is divided into non-overlapping blocks of two consecutive pixels. Then, by a series of reversible logic circuits, we calculate the difference value based on the values of the two pixels in each block and classify it as one of a set of continuous ranges. The secret image and operator information are embedded in the cover image by using the new obtained difference value to replace the original one. The number of bits of secret image that can be embedded in a block is determined, and the number of bits of operator information is decided by the range of the difference value belongs to. Moreover, when the embedded data is extracted from a stego image, it is not necessary to refer to the original cover image. The performance of the proposed scheme is based on the analysis of several categories of simulation results, such as visual quality, capacity, and robustness.

## Introduction

In recent decades, an increasing number of researchers have invested in the field of quantum image processing. Firstly, Vlasov^1^ proposed a method of recognizing orthogonal images. Later, G. Beach *et al*.^2^ showed that quantum algorithms like Grover algorithm^3^ can be used for image processing tasks. And then, the investigations about capturing and storing digital image on a quantum computer were explored. There are already a series of quantum representation models, such as Qubit Lattice^4,5^, Real Ket^6^ and so on. Among them, flexible representation of quantum images (FRQI)^7^ and a novel enhanced quantum representation of digital images (NEQR)^8^ are widely adopted. Then, on the basis of FRQI and NEQR, researchers have contributed to quantum image processing algorithms and applications, such as quantum image translation^9,10^, quantum image scaling^11–15^, quantum image feature extraction^16^, quantum image matching^17–19^, and so on^20^.

Especially quantum information hiding strategies have aroused considerable research interest, including quantum image steganography and quantum image watermarking. Like with classical steganography^21^, which has been thoroughly studied, quantum image steganography aims to make secret data concealed in the cover image undetectable by external observers.

Since the least significant bit (LSB) method hides secret information to a cover image in a simple way, it gained more and more researchers’ attention. In 2015, Jiang *et al*. proposed two quantum image steganography schemes based on moire patterns^22^ and LSB^23^, respectively. In 2016, Sang *et al*.^24^ constructed a scheme in which quantum color image is the cover image. Using basic gates, Miyake *et al*.^25^ designed quantum circuits to achieve the aim of embedding secret information. And Heidari *et al*.^26^ investigated three methods to embed the secret data to red-green-blue channels. Furthermore, in 2017, Heidari *et al*.^27–30^ also proposed some LSB based methods to protect copyright. Zhou *et al*.^31^ proposed a scheme that includes three processes of extension, scrambling and embedding. A scheme based on embedding color watermark image is proposed by Li *et al*.^32^. Zhou *et al*.^33^ proposed a watermarking scheme adopting new scrambling transformation in 2018.

To improve the performance of robustness of the existing quantum steganography algorithm, we proposed a quantum steganography scheme based on pixel value differencing (PVD). The concept of PVD was first proposed in ref.^34^. Because the human visual system has such a characteristic that human eyes are more sensitive to pixel modification of the smooth area of an image than the edge one, the amount of modification that each pixel of the digital image can tolerate is different. That is, without causing perceptible sensory distortion, each pixel can embed a different number of secret bits. But the amount of modification per pixel is uniform in the LSB steganography algorithm. The LSB steganography algorithm does not consider the character and ignores the edge effect of the image, so the algorithm performance is general. Considering the image edge effect and human visual system characteristics, each pixel in the digital image can be tolerated different bits in the PVD algorithm.

The proposed steganography scheme divides the cover image into blocks of two pixels that do not overlap. If the pixel difference of the pixel block is small, it indicates that this block is in the smoothing region. That means the human eye is more sensitive to it, and only less secret data can be hidden. Conversely, if the pixel difference value of block is large, it indicates that the block is located in the edge region of the image. The human eye is less sensitive to it, and more secret information can be embedded. The secret information includes a secret image and operation information, wherein the operation information may include operator information, operation time, etc., that can be used to trace the secret image.

## Preliminaries

### The novel enhanced quantum representation for digital images (NEQR)

NEQR model^8^ uses the basis state of a qubit sequence to store the grayscale value of pixels in the image. Therefore, two entangled qubit sequences are used in NEQR to store the whole image. For a 2^*n*^ × 2^*n*^ quantum image with ranged of 2^*q*^, the representative expression of NEQR image is expressed as follows:

1 $$|I\rangle =\frac{1}{{2}^{n}}{\sum }_{Y=0}^{{2}^{n}-1}{\sum }_{X=0}^{{2}^{n}-1}|f(Y,X)\rangle \otimes |YX\rangle =\frac{1}{{2}^{n}}{\sum }_{Y=0}^{{2}^{n}-1}{\sum }_{X=0}^{{2}^{n}-1}\mathop{\mathop{\otimes }\limits_{k=0}}\limits^{q-1}|{C}_{YX}^{k}\rangle \otimes |YX\rangle $$

where *q*-qubit sequence $|{C}_{YX}^{k}\rangle =|{C}_{YX}^{q-1}\cdot \cdot \cdot {C}_{YX}^{1}{C}_{YX}^{0}\rangle$ encodes the grayscale value *f*(*Y*, *X*) of the corresponding pixel (*Y*, *X*) and $|YX\rangle =|{y}_{n-1}\cdot \cdot \cdot {y}_{1}{y}_{0}\rangle |{x}_{n-1}\cdot \cdot \cdot {x}_{1}{x}_{0}\rangle$ represents the position information in vertical and horizontal directions.

### The pixel value differencing method (PVD)

PVD method is first proposed in ref. ^34^, in which, a cover image is partitioned into non-overlapping blocks of two consecutive pixels, say ${p}_{i}$ and ${p}_{i+1}$. A difference value *d* is calculated from the values of the two pixels by subtraction operation, which may be in the range from −255 to 255. Only consider the absolute values of ${d}_{i}$ and classify them into a number of contiguous ranges, called ${R}_{i}$, where $i=1,2\ldots n$. The number of bits can be embedded in a pixel pair is decided by which range the difference value belongs to. The difference value then is replaced by a new value to embed the bits of the secret information. This method provides an easy way to produce a more imperceptible result than those yielded by simple least significant bit (LSB) replacement methods. And also, the embedded secret information can be extracted from the stego image without referencing the original cover image.

### Reversible logic circuits

In this section, a series of reversible logic circuits is predefined to accomplish the PVD method. More details are described as follows.

#### Cyclic shift transformation (CS)

**Figure 1:**
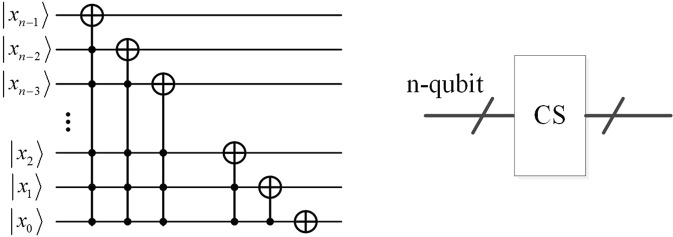
The cyclic shift transformation for one unit. It is consisting of a series of Controlled-NOT gates, and the number of control qubits is from n-1 to 0. The simplified module is shown on the right.

The cyclic shift is the realization of the position shifting transformation that was proposed in ref. ^35^. The reversible logic circuit is illustrated in Fig. 1, and its function can be expressed as

2 $$|{x}_{n-1}\,{x}_{n-2}\ldots {x}_{2}\,{x}_{1}\,{x}_{0}\rangle \to |({x}_{n-1}\,{x}_{n-2}\ldots {x}_{2}\,{x}_{1}\,{x}_{0}+1){\rm{mod}}\,{2}^{n}\rangle $$

where *n* is the number of qubits in cyclic shift transformation.

Therefore, when we move the image to the left by one unit, the pixels will be transformed from $|f(Y,X)\rangle$ to $|f(Y,X+1)\rangle$.

#### Plain adder module (ADDER)

The addition of two qubit sequences $|A\rangle$ and $|B\rangle$ by plain adder module is used in the proposed scheme that writes the result of the computation into one of the input sequences, i.e.

3 $$|A,B\rangle \to |A,A+B\rangle .$$

**Figure 2:**
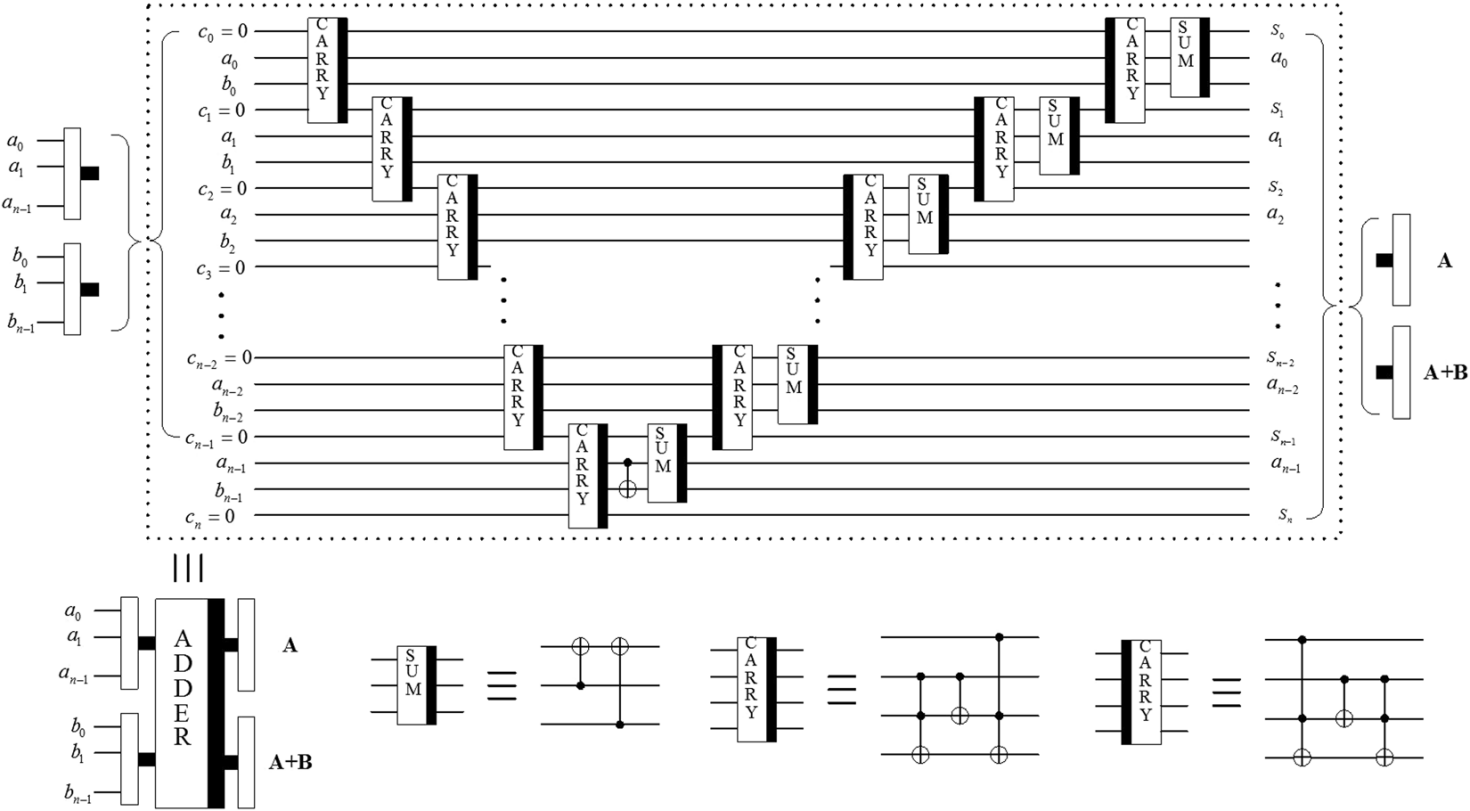
Circuit realization of plain adder module ADDER. The circuit in the dashed box implements the addition of two qubit sequences, and the three basic modules are illustrated below. The simplified module of ADDER is also given together.

The reversible logic circuit of ADDER module^36^ is shown in Fig. 2.

#### Calculate the absolute value (CAV)

**Figure 3:**
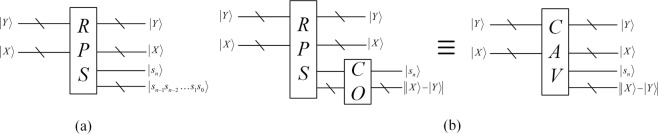
(**a**) Simplified circuit module of RPS (**b**) Module of calculating the absolute value

Zhou *et al*.^15^ designed a reversible parallel subtractor (RPS) through a series of basic modules. The simplified circuit module of RPS is illustrated in Fig. 3(a). There are two n-qubit inputs X and Y, where $|X\rangle =|{x}_{n-1}{x}_{n-2}\ldots {x}_{1}{x}_{0}$ and $|Y\rangle =|{y}_{n-1}{y}_{n-2}\ldots {y}_{1}{y}_{0}\rangle$. And the output $|S\rangle =|{s}_{n}{s}_{n-1}{s}_{n-2}\ldots {s}_{1}{s}_{0}\rangle$ is the result of $|X-Y\rangle$. It is worth noting that the highest qubit *s*_*n*_ is the sign bit. When *s*_*n*_ is equal to 1, Y is greater than X and $|S\rangle$ is the complement code of difference value. When *s*_*n*_ is equal to 0, it means that X is greater than Y and $|S\rangle$ is difference value.

To calculate the absolute difference value, the reversible logic circuit implementing the complement operation (CO) is constructed. The integrated CAV module is shown in Fig. 3(b). For more details, please refer to ref.^15^.

#### Quantum divider

**Figure 4:**
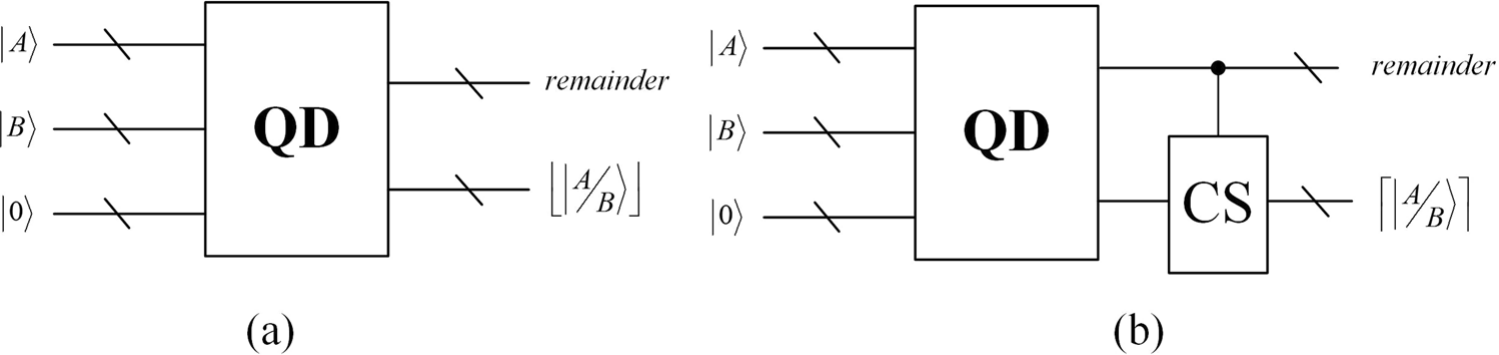
Quantum divider circuits (**a**) Round down quotient module (**b**) Round up quotient module. When the remainder is not equal to zero, the circuit module CS is adopted to round up the quotient.

The reversible logic circuit for implementation of division operation based on restoring division algorithm was proposed in^37^. Figure 4(a) illustrates a compendious quantum divider module. The inputs are $|A\rangle =|{a}_{n-1}{a}_{n-2}\ldots {a}_{1}{a}_{0}\rangle$, $|B\rangle =|{b}_{n-1}{b}_{n-2}\ldots {b}_{1}{b}_{0}\rangle$ and *n* ancillary qubits with an initial value of $|0\rangle$. The output contains the value of quotient that is rounded down. And we add a controlled cyclic shift operation mentioned in subsection 3.1 to the QD to acquire the round up quotient.

## Proposed Scheme

The secret image and the information of operator are embedded in cover image in the proposed traceable steganography scheme based on pixel value differencing. Wherein, the secret image is embedded regardless of the difference of the pixel values, and the operator information is embedded with different qubit numbers according to the level of the pixel value difference. Traceability of secret information is realizing by extracting operator information. More details are described next.

### Quantization of differences of gray values of two-pixel blocks

**Figure 5:**
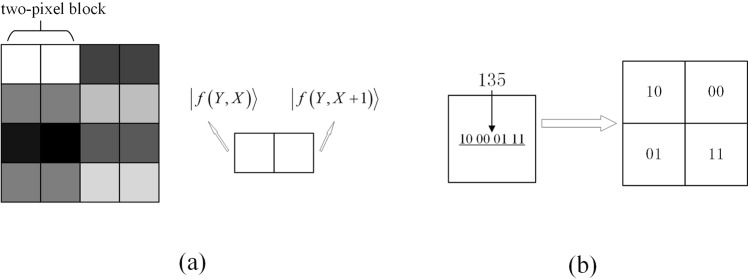
Two examples. (**a**) Example of the non-overlapping two-pixel blocks. The two pixels of same gray color in the image are the non-overlapping block. (**b**) Example of decomposing eight bits to four two-bits. The eight bits are divided into pairs, and then orderly filled into four positions.

Through analysis of the PVD method as described in subsection 2.2, it is known that the difference value *d* of $|f(Y,X)\rangle$ and $|f(Y,X+1)\rangle$ can be calculated by the reversible logic circuit CAV module so that we can partition the difference values in ranges ${R}_{i}$. Firstly, the way of dividing the square cover image into two-pixel blocks runs through all the rows of image in a consecutive and non-overlapping manner, and an example is shown in Fig. 5(a).

In general, small difference value indicates that the two-pixel block is in a smooth area, whereas a large difference value of a two-pixel block is corresponding to an edge area. The blocks in edge areas may, as mentioned previously, tolerate larger changes of pixel values than those in the smooth areas. Therefore, more information is embedded in edge areas than smooth areas.

**Figure 6:**
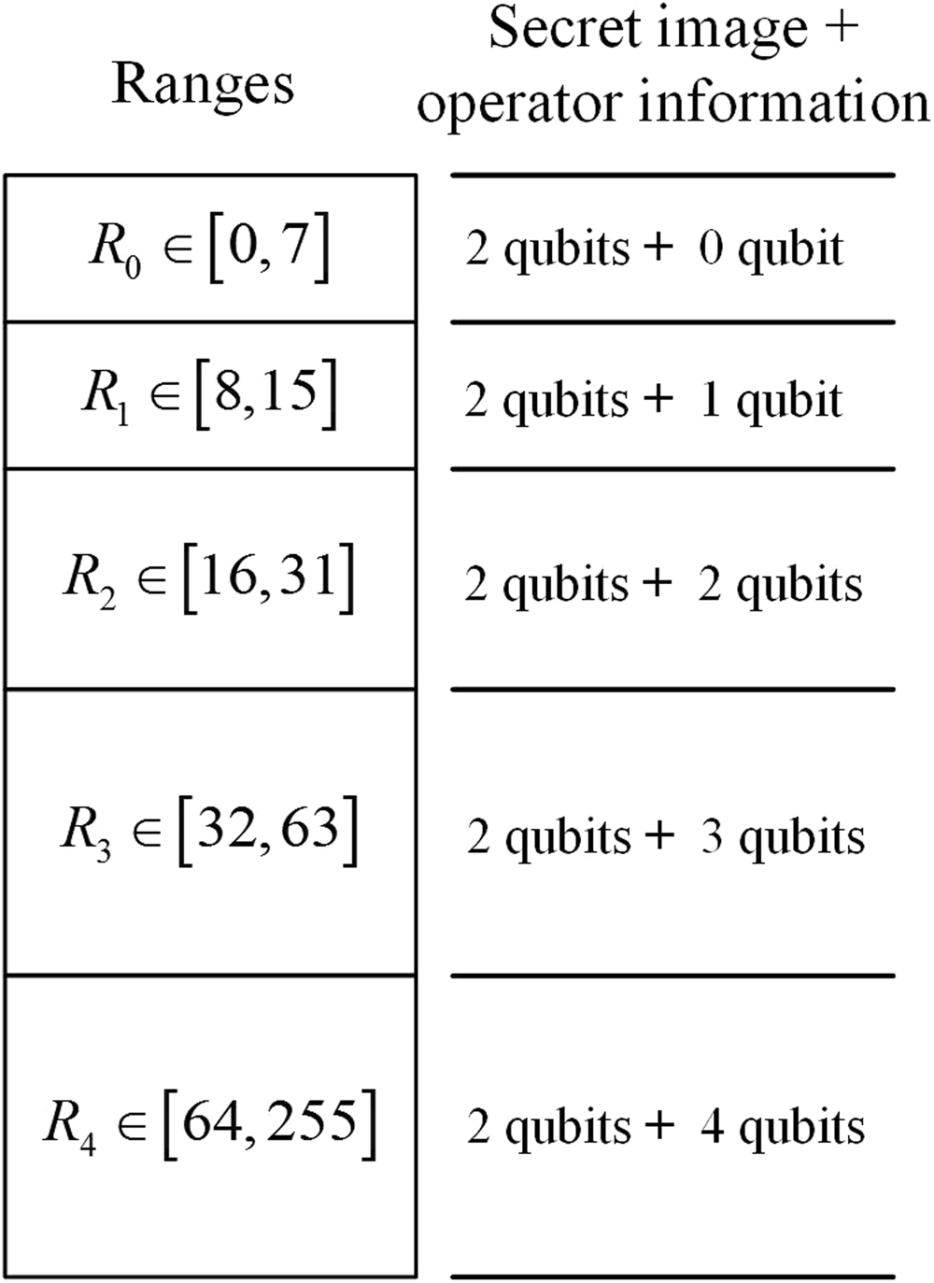
Ranges of the difference value

Specifically, $|d\rangle$ is classified into a set of continuous ranges, say ${R}_{i}$ where $i=0,1\ldots 4$ as shown in Fig. 6. The proposed scheme is based on selecting the range widths of 8, 8, 16, 32, and 192, which partition the total range of [0,255] into [0, 7], [8, 15], [16, 31], [32, 63], [64, 255]. Two qubits in secret image are embedded in the pair pixels in every range. In contrast, the number of embedded operator information qubits varies with the difference value. That is, when the difference value is at ${R}_{0}$, it is not embedded. When it is at ${R}_{1}$, one qubit is embedded. By this analogy, when the difference value is at ${R}_{4}$, four qubits are embedded.

### Data embedding

We propose a traceable steganography scheme utilizing the NEQR model, which hides a secret grayscale image and a bit stream (operator information) into a cover grayscale image. The size of secret image and cover image is ${2}^{n-1}\times {2}^{n-2}$ and ${2}^{n}\times {2}^{n}$, respectively. In order to correspond to the number of difference values, the secret image with ${2}^{n-1}\times {2}^{n-2}$ and 8 bits grayscale is expanding to an image with ${2}^{n}\times {2}^{n-1}$ and 2 bits grayscale. Figure 5(b) illustrates an example about how to decompose eight bits sequence into four two-bit sequences.

Then, the cover image and the decomposed secret image are transformed into quantum images $|C\rangle$ and $|S\rangle$, respectively. The representations can be expressed in Eq. (4):

4 $$\begin{array}{rcl}|C\rangle  & = & \frac{1}{{2}^{n}}{\sum }_{Y=0}^{{2}^{n}-1}{\sum }_{X=0}^{{2}^{n}-1}|f(Y,X)\rangle |YX\rangle =\frac{1}{{2}^{n}}{\sum }_{Y=0}^{{2}^{n}-1}{\sum }_{X=0}^{{2}^{n}-1}\mathop{\mathop{\otimes }\limits_{i=0}}\limits^{7}|{C}_{YX}^{i}\rangle |YX\rangle \\ |S & = & \frac{1}{{2}^{\frac{2n-1}{2}}}{\sum }_{Y=0}^{{2}^{n}-1}{\sum }_{X=0}^{{2}^{n-1}-1}|f(Y,X)|YX\\  & = & \frac{1}{{2}^{(2n-1)/2}}{\sum }_{Y=0}^{{2}^{n}-1}{\sum }_{X=0}^{{2}^{n-1}-1}|{S}_{YX}^{1}{S}_{YX}^{0}\rangle |YX\rangle \end{array}$$

and the difference values of the cover image $|d\rangle$ are written as below:

5 $$|d\rangle =\frac{1}{{2}^{(2n-1)/2}}{\sum }_{Y=0}^{{2}^{n}-1}{\sum }_{X=0}^{{2}^{n-1}-1}\mathop{\mathop{\otimes }\limits_{i=0}}\limits^{7}|{d}_{YX}^{i}\rangle |YX\rangle .$$

**Figure 7:**
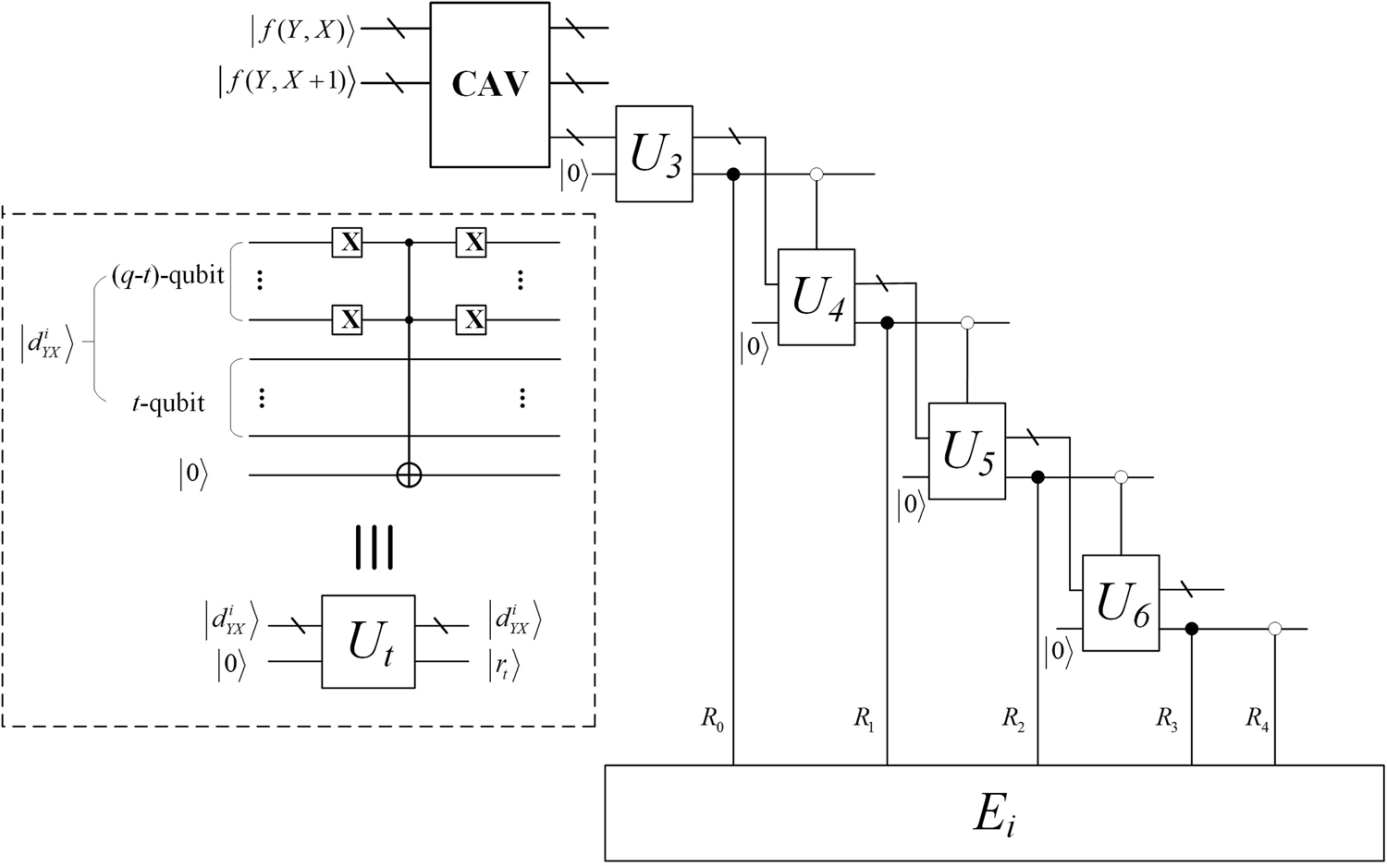
Circuits of classifying the difference value. In the dotted box, after flipping the highest q-t qubits using the X gates, a Controlled-NOT gate and an auxiliary qubit are used to assess if the highest q-t qubits are all zero, and then restore the original value with the same number of X gates. Four simplified module U_t_ are employed to divide the absolute difference value into five ranges, that is, R_0_, R_1_, R_2_, R_3_ and R_4_. For different ranges, different E_i_ will be adopted. (The module E_i_ is described below)

To realize the partition of $|d\rangle$, based on thresholds the pixel value differences comparison operation ${U}_{t}$ are proposed. The module of classification and the corresponding circuit is illustrated in the dotted box of Fig. 7. The number of t is changed according to the upper bound of ranges to be compared. Firstly, it is utilized to compare $|d\rangle$ with $|00000111\rangle$ that is the upper bound of first range and the parameter ${\rm{t}}$ is equal to 3 at this time. If the output $|{r}_{i}\rangle$ is equal to 1, it means that $|d\rangle$ is less than 8. That is, the range of $|d\rangle$ is ${R}_{1}$. If the output $|{r}_{i}\rangle$ is equal to 0, $|d\rangle$ is compared with the next upper bound. Similarly, all $|d\rangle$ can be partitioned in accordance with ${U}_{t}$, where $t=3,4,5,6$. For realizing the pixel value differencing and the determination of ranges, the whole reversible logic circuit is designed as shown in Fig. 7.

**Figure 8:**
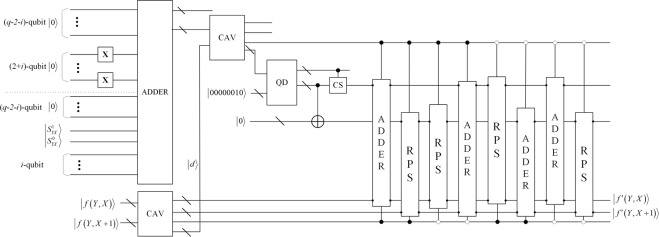
The embedding circuit E_i_. i is the level of difference value range. Distinguishingly, when i = 0, the number of X gate is set to zero. The first ADDER module adds the lower bound of the range to the secret information qubits, and the result is subtracted from the pixel value difference. After dividing by 2 using the module QD, the round up or down quotient is added or subtracted from the original pixel values according to the constraint of Eq. (7).

**Figure 9:**
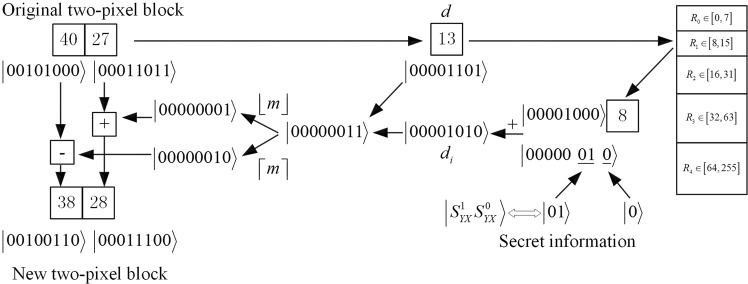
An illustration of the data embedding. The gray values of a sample two-pixel block are assumed to be (40, 27). The difference value is 13, which is in the range of 8 through 15. Therefore, the difference value is in the range of R_1_, which means that three qubits are embedded in cover image, that is, the value of $|{S}_{YX}^{1}{S}_{YX}^{01}\rangle$ and operator information qubit is $|01\rangle$ and $|0\rangle$, respectively. It is added to the lower bound value 8 of R_2_, resulting in a new difference value 10. Next, the new pixel values (38, 28) are obtained by the operational criterion in Eq. (7).

Since the number of embedded qubits in each range is confirmed, a new difference *d*′ then is computed by:

6 $$d^{\prime} ={l}_{k}+{b}_{i}$$

where ${b}_{i}$ is the denary value of embedded qubits, that is consisted of 2-qubit in secret image and i-qubit in operator information. And the value ${b}_{i}$ is in the range from 0 to ${u}_{k}-{l}_{k}$, hence the value of *d*′ is in the range from ${l}_{k}$ to ${u}_{k}$, where ${u}_{k}$ and ${l}_{k}$ represent the upper and lower bounds of the range ${R}_{k}$. According to the previous discussions, if we replace ${\rm{d}}$ with *d*′, the resulting changes are presumably unnoticeable to the observer. Through a series inverse calculation, ${b}_{i}$ is embedded in two-pixel block with pixel values of ${g}_{j}$ and ${g}_{j+1}$, respectively. The function is defined to be:

7 $$|f({g}_{i},{g}_{i+1})\rangle =\{\begin{array}{c}|{g}_{i}+\lceil m/2\rceil \rangle ,\,|{g}_{i+1}-\lfloor m/2\rfloor \rangle \,if\,{d}_{i}^{^{\prime} } > {d}_{i}\,and\,{g}_{i}\ge {g}_{i+1}\\ |{g}_{i}-\lfloor m/2\rfloor \rangle ,\,|{g}_{i+1}+\lceil m/2\rceil \rangle \,if\,{d}_{i}^{^{\prime} } > {d}_{i}\,and\,{g}_{i} < {g}_{i+1}\\ |{g}_{i}-\lceil m/2\rceil \rangle ,\,|{g}_{i+1}+\lfloor m/2\rfloor \rangle \,if\,{d}_{i}^{^{\prime} }\le {d}_{i}\,and\,{g}_{i}\ge {g}_{i+1}\\ |{g}_{i}+\lceil m/2\rceil \rangle ,\,|{g}_{i+1}-\lfloor m/2\rfloor \rangle \,if\,{d}_{i}^{^{\prime} }\le {d}_{i}\,and\,{g}_{i} < {g}_{i+1}\end{array}$$

where $|m\rangle =||d^{\prime} -d|\rangle$. The corresponding reversible logic circuit, that is module ${E}_{i}$, is demonstrated in Fig. 8. To assist one better comprehend the embedded procedure, an example of data embedding is illustrating in Fig. 9.

### Extraction procedure

The extraction procedure is as follows:

1. The stego image is divided into adjacent two-pixel blocks $({y}_{j}^{^{\prime} },{y}_{j+1}^{^{\prime} })$ according to the same partition method in the embedding step.
2. Calculate the difference value by Eq. (8), and determine the range ${R}_{i}$ in which it is located.
  8 $${d}_{j}^{^{\prime} }=|{y}_{j}^{^{\prime} }-{y}_{j+1}^{^{\prime} }|$$
3. Extract the secret qubits from ${d}_{j}^{^{\prime} }$ by:
  9 $${b}_{i}={{d}^{\text{'}}}_{j}-{l}_{i},$$
  where the last $(2+i)$ qubits of ${b}_{i}$ are the target.
4. Reorganize all extracted qubits to get the original secret image and operator information.

**Figure 10:**
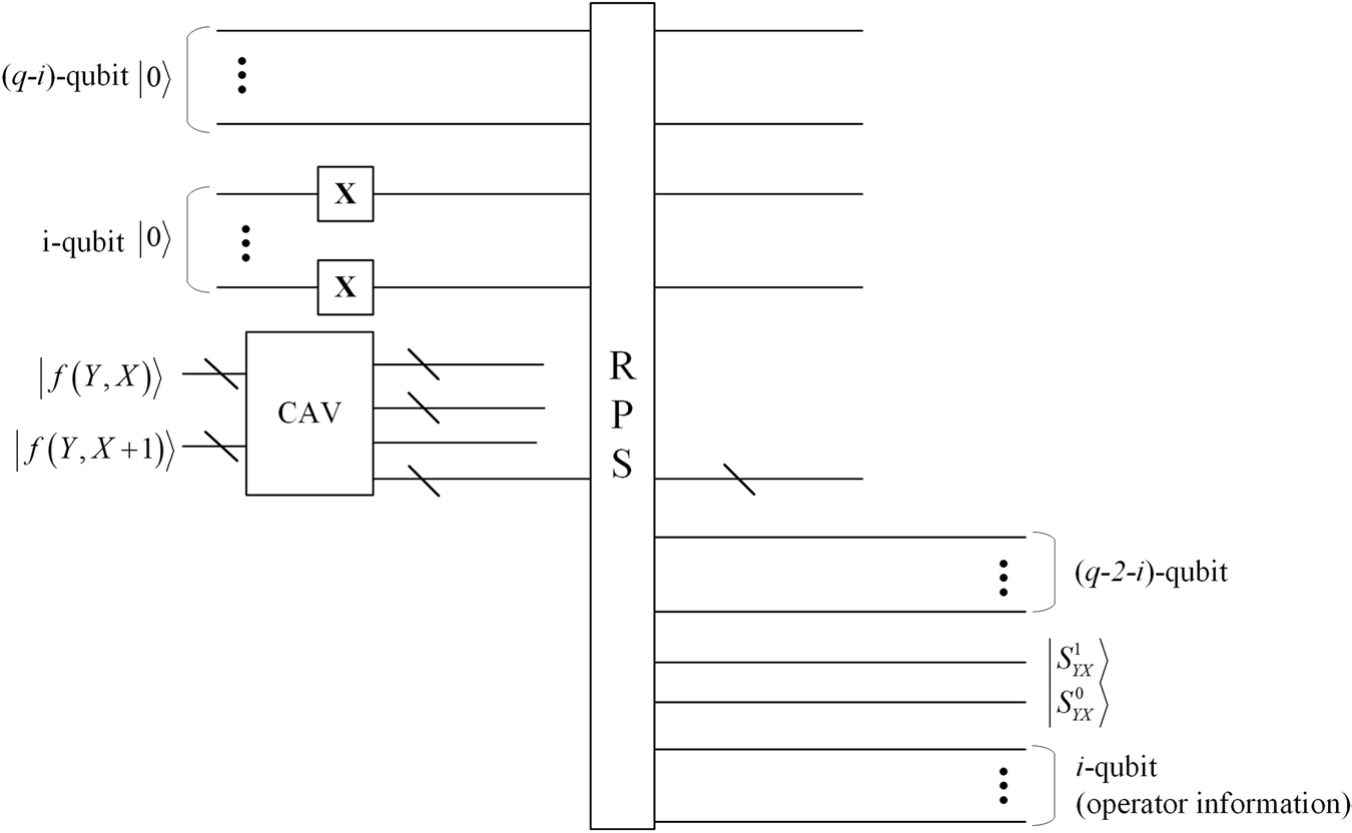
The extraction circuit. The last i + 2 qubits of output of RPS module which calculate the difference value of ${d}_{j}^{^{\prime} }$ and the lower bound of the range are the target qubits, wherein last i qubits are the operator information and other two qubits are the value of secret image.

So far, the accurate extraction of secret image without the original cover image is achieved. Simultaneously, we also extract operation information about the secret image. The extraction circuit is given in Fig. 10.

## Time Complexity

In order to calculate the time complexity of quantum image processing algorithms, usually a basic gate is considered. For all complex unitary operations on many qubits can be expressed as compositions of all one-qubit quantum gates and the two-qubit quantum CNOT gate^38^. Therefore, the time complexity of any one-qubit gates and two-qubit gates is taken as a basic unit. The circuits of classification are dividing into one CAV module and (q-4) U_t_ modules. And in the embedding circuit shown in Fig. 8, there are including two CAV modules, five ADDER modules, four RPS modules, one QD module and one CS module.

For a q-qubits CAV module, it contains a CO module that includes q CNOT gates and q q-CNOT gates and a RPS module which complexity is $7q-2$. Thus, the complexity of CAV module is:

10 $$q+q\times (12q-11)+7q-2=12{q}^{2}-3q-2$$

And the q-qubit ADDER module is composed by 2q carry module (which circuit complexity is 3), q sum modules (which circuit complexity is 2) and an additional CNOT gate. So the circuit complexity of ADDER modules is:

11 $$2q\times 3+q\times 2+1=8q+1$$

In addition, a q qubits QD module^37^, the circuit complexity is $3{q}^{3}+6{q}^{2}+q$. Reference^39^ points out that a q-CNOT gate is equivalent to $(2q-1)$ Toffoli gates and 1 CNOT gate with adequate ancillary qubits, and one Toffoli gate can be simulated by six CNOT gates. So the circuit complexity of CS module is:

12 $${\sum }_{i=0}^{q-1}[6\times (2i-1)+1]=6{q}^{2}-11q$$

Thus, the complexity of embedding circuit is

13 $$\begin{array}{c}2\times (12{q}^{2}-3q-2)+5\times (8q+1)+4\times (7q-2)\\ +(3{q}^{3}+6{q}^{2}+q)+(6{q}^{2}-11q)\\ =\,3{q}^{3}+36{q}^{2}+52q-7\end{array}$$

For the Fig. 7, the complexity is:

14 $$\begin{array}{c}(12{q}^{2}-3q-2)+{\sum }_{i=3}^{q-1}[2(q-t)+6(q-t)]\\ =\,16{q}^{2}-27q+30\end{array}$$

Thus, the total circuit complexity of data embedding is $3{q}^{3}+53{q}^{2}+25q+23$, that is, ${\rm O}({q}^{3})$.

For the extraction procedure, the circuit of classification is same as the embedding procedure which is given in Eq. (14) and the extraction circuit consists of one CAV module and one RPS module. Therefore, the circuit complexity is:

15 $$\begin{array}{c}(16{q}^{2}-27q+30)+(12{q}^{2}-3q-2+7q-2)\\ =\,28{q}^{2}-23q+26\end{array}$$

that is $${\rm O}({q}^{2})$$.

We can see from the above, the complexity of embedding and extraction procedures is ${\rm O}({q}^{3})$ and ${\rm O}({q}^{2})$, respectively. This is only related to the qubits representing the gray scale. Compared with the complexity related to image size in the classical counterpart, our algorithm has a larger improvement than the classical algorithm.

Furthermore, we compare the time complexity of proposed scheme with other quantum information hiding schemes, in which the image size is ${2}^{n}\times {2}^{n}$ and gray scale is ${2}^{q}$. The results are enumerated in Table 1, we can see that the complexity of other schemes is related to the size of image, but the proposed scheme is related to the gray scale. Therefore, different from the complexity of other schemes that varies with image size, the complexity of the proposed scheme does not increase as the image gets larger.

**Table 1:** The comparison of complexity.

Scheme	The time complexity
Reference^20^	$19n+8$
Reference^25^	$3n+2$
Reference^26^ -first method	$18{n}^{2}+42n+94$
Reference^26^ -second method	$18{n}^{2}+90n+504$
Reference^26^ -third method	$18{n}^{2}+98n+166$
Proposed scheme	$3{q}^{3}+53{q}^{2}+25q+23$

## Simulation Experiments and Discussion

In order to evaluate the proposed scheme comparing with the existing literature, in this section, simulations of the properties are demonstrated. All the simulations are based on a classical computer equipped with software Matlab R2014b. The cover images used in the simulation experiments are “Male”, “Peppers” “Sailboat on lake” and “Airplane” with size of $256\times 256$. Besides that, in order to facilitate the traceability, the operator information is a bit stream that full of quantum representation of the text “Quantum Text and Quantum Image”^30^.

### Invisibility

#### The histogram analysis

Image histogram can be considered as a visualized tool for evaluating the visual effects caused by image steganography on cover images. The image histogram is a statistic of the gray level distribution in the image, that counting all the pixels in the image according to the gray value. Wherein, the abscissa is a gray level, and the ordinate is a frequency at which the gray level appears. By comparing the histogram graphs of two images one can judge whether the images similar or not. In image steganography algorithms, more similarity can be observed between the histogram of cover image and corresponding stego image, more invisibility can be obtained after the image steganography scheme manipulated.

**Figure 11:**
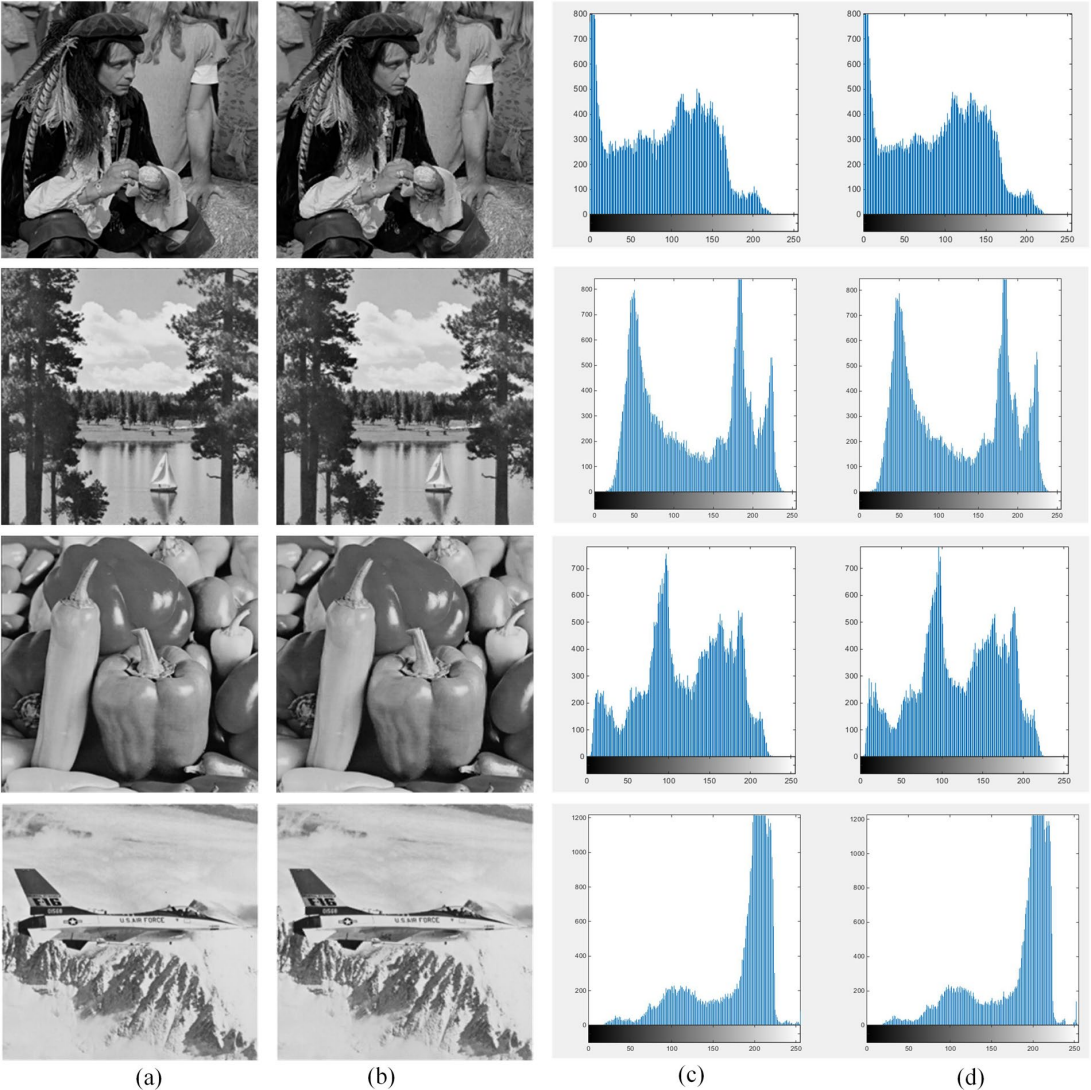
Each column from left to right is (**a**) original images (**b**) stego images (**c**) original image histogram graphs (**d**) stego image histogram graphs.

Figure 11 indicates the histogram graphs of the six original images and the histogram graphs of their corresponding stego images where the image “Male” with size of $128\times 64$ is considered as the secret image. According to the histogram graphs, it can be seen that the stego images are in good agreement with the original ones.

#### The peak signal-to-noise ratio (PSNR)

Since the peak signal to noise ratio (PSNR) is generally used to evaluate the quality of the stego image, we adopt that to evaluate fidelity of our steganography scheme. The PSNR is defined as follows:

16 $$PSNR=20{\log }_{10}(\frac{MA{X}_{P}}{\sqrt{MSE}})$$

Herein, $MA{X}_{P}$ is the maximum pixel value of the cover image, i.e., 255. MSE is defined as the mean squared error for two $m\times n$ images P and Q, where P and Q are associated with the stego image and the cover image, respectively.

17 $$MSE=\frac{1}{mn}{\sum }_{i=0}^{m-1}{\sum }_{j=0}^{n-1}[{(P(i,j)-Q(i,j))}^{2}]$$

The simulation results of PSNR are shown in Table 2, in which frequently-used images are selected as cover images, i.e., “Peppers”, “Sailboat on lake” and “Airplane”. Although the LSB based methods presented by Heidari *et al*.^27,29,30,40^ has higher PSNR, our proposed algorithm also achieves a satisfactory visual quality from invisibility analyses.

**Table 2:** The PSNR for the proposed scheme.

Cover image	Secret image	PSNR
Peppers	Airplane	42.65
Sailboat on lake	Peppers	41.31
Airplane	Male	42.26

### Capacity

The capacity of quantum steganography scheme is defined as the ratio of the number of secret qubits and the number of cover pixels. Thus, the proposed scheme’s capacity is given as follows:

18 $$\begin{array}{c}C=\frac{the\,num.of\,\sec \,retqubits}{thenum.of\,{\rm{cov}}\,erimagepixels}\\ =\,\frac{q\times {2}^{n-1}\times {2}^{n-2}+m}{{2}^{2n}}=\frac{8\times {2}^{n-1}\times {2}^{n-2}+m}{{2}^{2n}}=s(bit/pixel)\end{array}$$

where m and q are the embedded qubits of operator information and the gray scale of secret image, respectively. Therefore, the capacity of the proposed scheme is s that is greater than 1.

### Robustness

#### Robustness performance under noise

Obviously, the secret image can be integrally extracted in a noise-free environment. However, the extraction procedure of the proposed scheme is not always carried out in a noiseless environment. The robustness of the proposed scheme under the salt and pepper noise is analyzed. Salt and pepper noises are applied with different density of from 0 to 0.15 into $256\times 256$ stego image “Peppers”. Peak signal-to-noise ratio (PSNR) is employed to evaluate the fidelity of the extracted secret image. The corresponding results from the stego images with noise are shown in Table 3. The table also shows the PSNR values of the scheme proposed in refs^22,25,27^. As can be seen from Table 3, the value of PSNR in our scheme is obviously higher than the other three schemes.

**Table 3:** PSNR of extracted image under the salt and pepper noise.

Scheme	Density		
	0.05	0.1	0.15
Reference^22^	15.03	12.15	10.20
Reference^25^	26.96	22.14	18.34
Reference^27^	—	31.30	30.14
Proposed scheme	41.90	38.92	37.39

#### Robustness performance under attack

Since pure LSB based methods are easy detected, it is vulnerable to steganalysis. Regular and Singular (RS) steganalytic technique, first proposed in ref.^41^, is very efficient in detecting the presence of a message in a gray image and to estimate its approximate size. The technique originated by analyzing the capacity for lossless data embedding in the LSB. Randomizing the LSB decreases the lossless capacity in the LSB plane, but it has a different influence on the capacity for embedding that is not constrained to one bit-plane. Thus, the lossless capacity turned out to be a very sensitive measure for the degree of randomization of the LSB plane. And the secret message length can be derived by inspecting the lossless capacity in the LSB plane.

In the proposed scheme, the differences of the gray values in the two-pixel blocks of the cover image are used as features to cluster the blocks into a number of categories of smoothness and contrast properties. Different amounts of data are embedded in different categories according to the degree of smoothness or contrast. Therefore, we have no significant change in the ratio of regular and singular groups compared to the original image. This means that RS technique cannot detect the embedded data in the cover image of the proposed scheme.

## Conclusion

A new and traceable quantum steganography scheme for embedding secret image and operation information into cover image without producing noticeable changes has been proposed. The scheme is based on pixel value differencing which follows image edge effects and human visual system characteristics well. Pixels located in the edge area of the image are embedded with more secret information, including operator information for traceable secret images. Secret image and operation information are embedded into cover image by replacing the difference values of the two-pixel blocks of the cover image with similar ones in which qubits of embedded data are included. It is worth mentioning that the extraction process is absolutely blind. Furthermore, by embedding data in each adjacent pair of signals of images, the steganography scheme can also be easily extended to efficiently carry content-related messages such as captions or annotations in quantum audio and video.

## Data Availability

All data needed to evaluate the conclusions are available from the corresponding authors upon reasonable request.
